# Physiological temperature during brain slicing enhances the quality of acute slice preparations

**DOI:** 10.3389/fncel.2013.00048

**Published:** 2013-04-23

**Authors:** Shiwei Huang, Marylka Y. Uusisaari

**Affiliations:** ^1^Computational Neuroscience Unit, Okinawa Institute of Science and Technology Graduate UniversityJapan; ^2^Optical Neuroimaging Unit, Okinawa Institute of Science and Technology Graduate UniversityJapan; ^3^Department of Neurobiology, Hebrew University of JerusalemIsrael

**Keywords:** acute slice preparation, cerebellum, cerebellar nuclei, mature animals

## Abstract

We demonstrate that brain dissection and slicing using solutions warmed to near-physiological temperature (~ +34°C), greatly enhance slice quality without affecting intrinsic electrophysiological properties of the neurons. Improved slice quality is seen not only when using young (<1 month), but also mature (>2.5 month) mice. This allows easy in vitro patch-clamp experimentation using adult deep cerebellar nuclear slices, which until now have been considered very difficult. As proof of the concept, we compare intrinsic properties of cerebellar nuclear neurons in juvenile (<1 month) and adult (up to 7 months) mice, and confirm that no significant developmental changes occur after the fourth postnatal week. The enhanced quality of brain slices from old animals facilitates experimentation on age-related disorders as well as optogenetic studies requiring long transfection periods.

## INTRODUCTION

In cellular neuroscience research, acute brain slice preparation is the work-horse method for investigating microcircuits, single neurons, and sub-neuronal processes (Andersen, 1981), despite the necessity of other approaches (e.g., using living animals) when behavioral effects or large-scale circuit dynamics are studied. Numerous efforts (Lipton et al., 1995; Richerson and Messer, 1995; Brahma et al., 2000; Moyer and Brown, 2002; Ye et al., 2006) to identify the best conditions for preserving neuronal health and network structure during slice preparation notwithstanding, visually guided experimental methods in brain slices, such as patch-clamp recordings remain challenging in adult or old animals.

Various essential studies have been rendered difficult or even impossible because of this limitation. For instance, it has become a grudgingly accepted fact that all *in vitro* work from the deep cerebellar nuclei (DCN) is limited to using young animals (Linnemann et al., 2006; Bagnall et al., 2009) and no *in vitro* data from animals older than 28–30 postnatal (P) days have been published (Uusisaari and Knöpfel, 2010; Person and Raman, 2011). Therefore, studies aiming at answering questions related to aging or neuro-degenerative diseases cannot be investigated in this key structure of the olivo-cerebellar circuitry. Another example is the expression of virally transfected proteins in optogenetic studies. Post-transfection times of weeks are often required to reach sufficient expression levels (Shimano et al., 2012); thus, it is essential to be routinely able to perform slice experiments using brains of animals older than at least 6 weeks.

In this short methodological report, we critically examine the common assumption that cooling brain tissue to near-freezing temperatures during the slice preparation procedures is requisite to obtain healthy slices. Surprisingly, we find that slices prepared at near-physiological (> +30°C) temperature are healthier than those prepared under conventional “ice-cold” (< +5°C) conditions. Importantly, in near-physiological temperatures, excellent cerebellar nuclear slices from mature and old mice can be routinely obtained and used for patch-clamp electrophysiology.

## METHODS

### SLICE PREPARATION

Mice of either gender pertaining to two age groups were used in this study: immature (age range P17– P30, strain C57BL/6J 6w, Charles River) and mature (age range 2.5 – 7 months, strain C57BL/6J 6w, Swiss Webster, or wild-type litter mates from the D1-GFP line (Valjent et al., 2009). All animals were treated in accordance with the Science Council of Japan Guidelines for Proper Conduct of Animal Experiments. All experiments were approved by the OIST Animal Resources Section.

Mice were anesthetized with isoflurane and decapitated. The cerebellum was separated from the forebrain by a coronal cut, removed from the skull, and immediately glued to a cutting stage immersed in artificial cerebrospinal fluid (ACSF) containing (in mM): 125 sodium chloride (NaCl), 2.5 potassium chloride (KCl), 25 glucose, 25 sodium hydrogen carbonate (NaHCO_3_), 1.25 monosodium phosphate (NaH_2_PO_4_), 2 calcium chloride (CaCl_2_), and 1 magnesium chloride (MgCl_2_), gassed with 5% CO_2_/95% O_2_. Slices (250 μm sagittal or 300 μm coronal) were cut with a Campden Ci 7000 smz microtome using ceramic blades (both from Campden Instruments) at an advance speed of 0.01–0.05 mm/s. Vertical vibration of the blade was manually tuned in accordance with the user manual, and was set to 0.1 – 0.3 μm. Bath temperature was kept within the desired range, as described for the experiments by adding warm or cold water into the external chamber of the slicer, and was monitored throughout the cutting procedure with a conventional mercury/glass thermometer. Slices were then transferred to a holding chamber filled with oxygenated ACSF at +34°C and allowed to recover for at least 0.5 h before use during the next 1–8 h. All chemicals were purchased from Sigma (Sigma-Aldrich).

### MULTI-ELECTRODE ARRAY (MEA) RECORDINGS

For estimating the lower bound count of superficial, healthy DCN neurons, we employed a perforated multi-electrode array (MEA) recording system MEA60-Inv-BC (MultichannelSystems, Reutlinger) with a 6 × 10 electrode arrangement and electrode spacing of 100 μm (MEA chip: 60pMEA100/30iR-Ti-gr). Each coronal slice containing DCN was cut into two hemispheric slices, and each of the hemi-slices was placed on the recording area of the chip so that the recording area covered as large as an area of the DCN as possible. Light suction was applied through the chip to secure contact between the slice and electrodes. For further support, a conventional slice anchor bar was placed on top of the slice. The chip chamber was perfused with oxygenated ACSF and kept at room temperature. Recordings performed at room temperature were commenced immediately after obtaining a signal from the electrodes and were discontinued ≤5 min after placing the slice on the chip, to prevent possible degradation effects. Spikes were detected from continuous 2-minute long recordings (acquired at 25 kHZ) using the MC_Rack software (MultichannelSystems, Reutlinger).

### PATCH-CLAMP ELECTROPHYSIOLOGY

In the recording chamber, slices were superfused with oxygenated ACSF (2 ml/min, at 34°C). Neurons were visualized using infra-red differential interference contrast video microscopy (using an Olympus BX51WI microscope) with a 40x water-immersion objective lens. For whole-cell patch-clamp recordings in current-clamp mode, borosilicate glass electrodes of 5–7 MΩ were filled with an internal solution containing (in mM): 140 potassium gluconate, 10 KCl, 10 Hepes, 10 ethylene glycol tetraacetic acid (EGTA), 4 MgATP, 0.4 NaGTP, 10 phosphocreatine, 8 biocytin (pH adjusted to 7.3 with KOH). Purkinje neurons were identified by their typical morphology and position within the cerebellar cortex. For DCN, the large glutamatergic projection neurons were identified as described in Uusisaari and Knöpfel (2011).

Seal resistance was >4 GΩ before breaking the seal and entering whole-cell patch-clamp configuration. Signals were amplified and low-pass filtered at 5 kHz using a Cornerstone BVC-700A amplifier (Dagan) and were recorded at 40 kHz using a custom interface written in Labview acquisition software (National Instruments). After obtaining whole-cell configuration, both DCN and Purkinje neurons were hyperpolarized with current injection (< -0.25 nA) to keep them at a holding voltage of -65 mV.

### BIOCYTIN STAINING AND SLICE IMAGING

After recording, pipettes were slowly withdrawn to preserve integrity of the cell membrane. Slices were placed in 4% paraformaldehyde in 100 mM phosphate buffer (PBS, pH 7.3) for 24–48 h. The staining procedure used in this study has been described in detail by Marx et al. (2012). Briefly, slices were rinsed several times in PBS prior to quenching endogenous peroxidase activity with 3% H_2_O_2_. After washing in PBS, slices were incubated in avidin-biotinylated horseradish peroxidase (Vectastain elite ABC kit, Vector labs) for 4–10 h. After incubation, slices were repeatedly rinsed in PBS, followed by a nickel-intensified diaminobenzidine reaction (Vector DAB Substrate kit, Vector labs). Slices were mounted using Mowiol-based medium (Sigma-Aldrich). Images of stained neurons were acquired using a Zeiss LSM 710 microscope and Zen 2011 software. For documenting the visual appearance of acute slices, DIC images from the microscope were acquired using an IO-data GV-USB2 video capture connector and software, and were enhanced for clarity using Adobe Photoshop, with care being taken not to introduce artifacts into the images.

### ANALYSIS

Electrophysiological data were analyzed using Matlab (MathWorks). Data are given as means ± standard error, and statistical significance was tested using Student’s *t*-test (*p* < 0.05 was considered significant). Input resistance (*R*_m_) was calculated from the amplitude of initial peak and steady state voltage deflection (for Purkinje neurons and DCN neurons, respectively) in response to a small (≤10 pA) negative current step. The membrane time constant (Tau) was estimated by exponential fitting, where the fit duration was 500 ms starting 2 ms after current pulse injection. This was done to avoid artifacts due to a voltage drop across the recording electrode (Major et al., 1994; Roth and Hausser, 2001). Instantaneous firing frequency was calculated by inverting inter-spike intervals measured during depolarizing current step injections, during which neurons fired an average of 25 Hz (Purkinje neurons) or 20 Hz (DCN neurons). For firing frequency accommodation, instantaneous firing frequency values were normalized on the first inter-spike interval value. Action potential (AP) shape was obtained from averaging the first spikes of +0.1 – +0.2 nA current injection steps. AP half-width (APHW) was defined as the duration of APs at half-maximal amplitude. AP threshold was defined as the *V*_m_ measured 0.5 ms before the peak in the second derivative of the waveform. Spike after-hyperpolarization (AHP) depth was quantified as the voltage difference between the AHP peak and spike threshold voltage. *I*_h_-like sag was analyzed in DCN cells by calculating the mean amplitude of depolarization seen from the peak of a hyperpolarizing response to steady-state conditions.

Multi-electrode array signals were analyzed channel by channel and detected spikes were sorted to 1–4 cells per channel using P2S2 package (Aminov, 2010). All ambiguities in detected cell count per channel was resolved toward the lower value, thereby providing a comparable lower-bound estimate for superficial healthy neurons available in slices. For statistical analysis we compared 23 slices (from seven mice) cut at cool temperature (< +5°C) and room temperature (+ 28°C) with 10 slices (from four mice) cut at warm temperatures (+30 - +34°C).

## RESULTS

**FIGURE 1:**
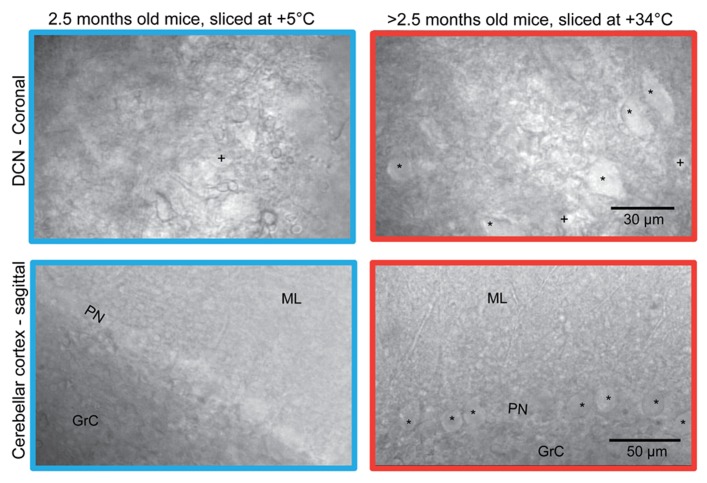
**Comparison of the surface of acute coronal (top) and sagittal (bottom) mice (strain C57BL/6J 6w) cerebellar slices prepared with ice-cold (left) and warm (right) preparation methods.** Patchable cells are indicated. All slices shown were prepared from mice aged 2.5 months, except for the sagittal slice cut at warm temperature (lower right) which was prepared from a 6-month old mouse. Note that in the warm-cut coronal slice, DCN cell bodies of both small (labeled “+”) and large (“*”) sizes are visible; in cold-cut coronal slices from old animals, most cells were small (labeled “+”). In cold-cut sagittal slices, hardly any healthy neurons in the Purkinje layer were found. In contrast, numerous patchable Purkinje neurons are easily seen in the warm-cut sagittal section. PN, Purkinje neuron layer; ML, molecular layer; GrC, granular cell layer.

Typically, when slices from mature (>2 months) animals are prepared by the conventional “ice-cold” method, i.e., by cooling the brain to nearly 0°C before and during slicing (Andersen, 1981), without trans-cardiac pre-perfusion (Moyer and Brown, 2002), the DCN slice surface appears uneven and damaged (**Figure 1**, top left). Therefore, even in the best slices, very few, if any, cell bodies can be found close enough to the surface to allow for visually guided patching (marked by “*”). Sagittal slices of cerebellar cortex, also prepared in the cold, have a somewhat cleaner appearance (**Figure 1**, bottom left), but it is laborious to find Purkinje neurons that do not show evidence of damage, such as dendritic and somatic swelling, visible nucleoli, or a wrinkled appearance.

However, when slices were cut in exactly the same manner, but with cutting solutions warmed to near-physiological temperatures (+34 – 35°C), slice appearance improved significantly (**Figure 1**, right panel). This was characterized by tens of apparently healthy DCN neurons (of several sizes, marked by “*” and “+” in the figure) at the surface of each coronal slice, and by nearly uninterrupted rows of healthy Purkinje neurons (marked by “*”) with smooth somata and proximal dendrites in sagittal slices.

**FIGURE 2:**
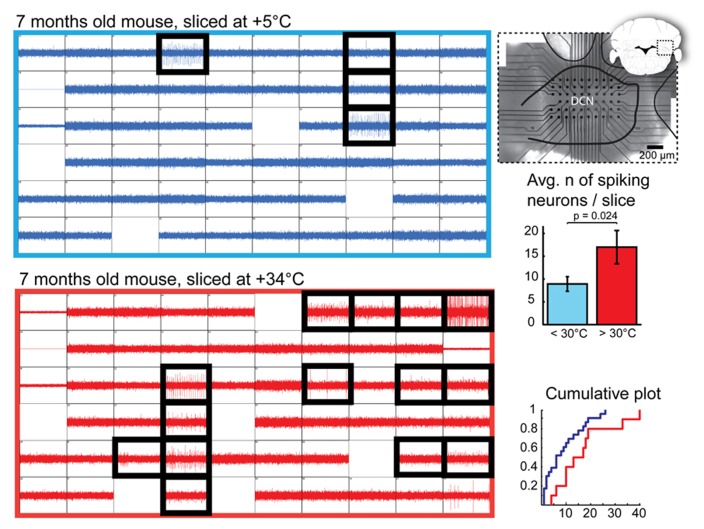
**Quantification of DCN slice healthiness with multi-electrode array recordings.** Left: representative recordings with 60 planar extracellular electrodes from 7-month old mice from DCN slices prepared at cold (top, blue) and warm (bottom, red) temperatures. Each grid square represents 1 s of continuous recording (vertical axis: 100 pA); blank squares are electrodes with degraded recording quality. Electrodes in which spiking cells were detected are indicated with black, thick squares. A micrograph combined with a schematic drawing of a coronal cerebellar slice (right top panel) shows positioning of the multi-electrode array within the DCN. Right, middle and bottom panels: Comparisons of average and cumulative sums of the numbers of spiking cells detected per slice in < +30°C (+5 to 28°C, blue) and +30 to +34°C (red) conditions shows that when slices are prepared at warm temperatures, the probability of finding patchable neurons is increased. *N* = 23 (seven animals) slices cut in cold conditions, 10 (four animals) in warm.

We next compared the number of viable DCN neurons on the surface of cold- and warm-cut slices. Most healthy neurons in the DCN are spontaneously active in the absence of synaptic input in slice preparations (Aizenman and Linden, 1999; Uusisaari et al., 2007). This allows us to utilize detection of spontaneously spiking units as an means to identify healthy DCN neurons. Thus, we recorded extracellular spiking activity in whole DCN slices prepared in a range of cold or room temperatures (+2 – +28°C) and warm (+30 – +34°C) temperatures and subsequently estimated the number of spiking (therefore, healthy) DCN cells found in each slice (**Figure 2**).

Even though we occasionally observed spiking neurons in cold-cut slices, as well as in those at room temperatures, in most of the warm-cut slices many more were evident (**Figure 2**, bottom right; mean, 8.9 ± 1.6 and 17.0 ± 3.6 cells per slice in cold and warm conditions, respectively; *p* = 0.023). In cold-cut slices, 9/23 slices (39%) had 10 or more spiking cells, whereas in warm-cut slices 10 more cells were found twice as often (8/10 slices). This result contradicts the notion that cold cutting temperature is better for neuronal health, and suggests instead that the opposite is true.

**FIGURE 3:**
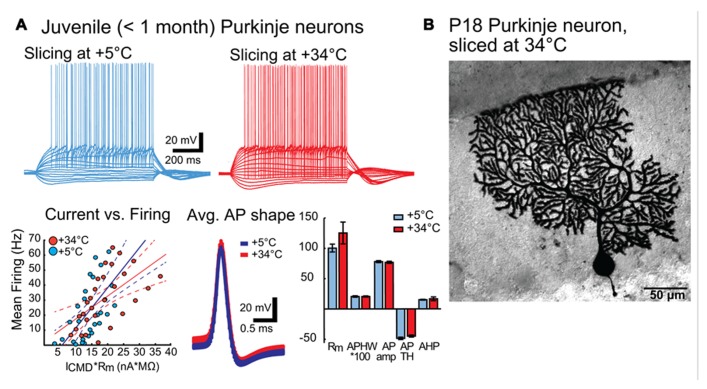
**(A)** Intrinsic properties of juvenile (P18 – 28) Purkinje neurons are not affected by preparation temperature. Top panel: representative traces of voltage responses to step current injections in PNs from slices prepared in cold (blue) and warm (red) solutions. No differences beyond normal Purkinje neuron (PN) variability were apparent. Bottom panel: Left: Current-versus-firing frequency relation (blue and red dots) with linear fits (solid line) and 95% confidence interval (dashed lines) show similar gains of AP firing. Middle: population average APs (from all PNs from slices cut in cold (blue) and warm (red) solutions) show nearly indistinguishable shapes. Width of the trace represents the mean *V*_m_ ± SEM. Right: Comparison of intrinsic membrane properties (input resistance (*R*m, MΩ), AP half-width (APHW, ms), AP amplitude (AP amp, mV), AP threshold (AP TH, mV), and AHP depth (mV) (right) show no statistically significant differences between cold (blue) and warm (red) preparation conditions (*R*_m_: cold: 100.4 ± 6.5 MΩ, warm: 125.2 ± 18.0 MΩ; APHW: cold: 0.21 ± 0.01 ms; warm: 0.21 ± 0.01 ms; AP amp: cold: 77.9 ± 1.6 mV; warm: 76.8 ± 1.7 mV; APTH: cold: -48.6 ± 2.0 mV; warm: -44.9 ± 1.7 mV; AHP: cold: 15.0 ± 0.7 mV; warm: 16.6 ± 3.1 mV. *P* value for each data group >0.05. N: cold-cut *n* = 5, warm-cut *n* = 5. *R*_m_, input resistance; AP, action potential; APHW, action potential half-width; *I*_CMD_, step command current; C_m_, estimated neuronal capacitance (see methods). For averaged AP shapes, the thickness of the trace signifies ± SEM. Note that for bar graphs in **A** (bottom right), values for APHW and *I*_h_-sag have been scaled (100× and 10×, respectively) for visual clarity. Also, note that in the left graph of the bottom panel, for correct comparison of current-to-firing relationships between neurons of possibly differing membrane resistances, the command current (*I*_CMD_) is scaled by multiplying by the measured *R*_m_ for each cell. **(B)**. Morphology of a Purkinje neuron from a warm-cut slice (mouse age P18) filled with biocytin during electrophysiological recording. Compared to cold-cut, biocytin-filled Purkinje neurons (Jaeger, 2001; Bekkers and Häusser, 2007), there was no evidence suggesting that dissection temperature affects dendritic structure.

To confirm that improved quality of the slice surface prepared in warm temperature was not negated by degraded neuronal physiology, we performed whole-cell patch-clamp recordings on Purkinje neurons prepared under both cold and warm conditions (**Figure 3**). Comparisons of intrinsic properties of juvenile (P18-28) Purkinje neurons in sagittal slices revealed no differences in terms of input resistance (*R*_m_), AP features (AP amplitude, half-width, threshold, after-hyperpolarization (AHP) amplitude) or current-to-firing relationship (**Figure 3A**). Furthermore, Purkinje neurons filled with biocytin and stained revealed no evidence of dendritic swelling or other signs of neuronal degradation (**Figure 3B**).

**FIGURE 4:**
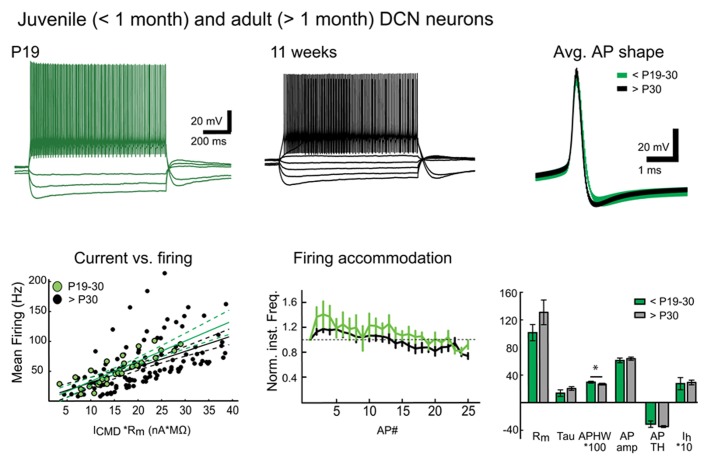
**Warm preparation temperature allows patch-clamp recordings from mature and old DCN neurons.** Top panel: Representative examples of voltage responses of DCN neurons to step current injections (two leftmost panels) as well as population average AP shapes in juvenile (less than P30) and mature (1–7 months old) animals are nearly indistinguishable. Bottom panel: Comparison of current-versus-firing (with linear polynomial fits and 95% prediction bounds), spike frequency accommodation (vertical bars denote frequency standard error values. All values are normalized to the first inter-spike interval firing frequency; a dashed line demarcates the starting value) and intrinsic membrane measures (*R*_m_ (MΩ), membrane time constant (Tau, ms), AP amplitude (AP amp, mV), AP threshold (AP TH, mV), AHP depth (AHP, mV), and *I*_h_-sag (mV)) reveal no statistically significant differences between juvenile and old DCN neurons (Values: *R*_m_: young: 101.5 ± 11.8 MΩ, old: 130.9 ± 17.9 MΩ; Tau, young: 13.6 ± 4.7 ms, old: 20.3 ± 2.7 ms; AP amp: young: 61.0 ± 3.4 mV; old: 63.6 ± 2.3mV; APTH: young: -48.6 ± 2.0 mV; old: -44.9 ± 1.7 mV; AHP: young: 24.0 ± 6.7 mV; old: 22.5 ± 1.2 mV; *I*_h_-sag: young: 27.2 ± 8.9 mV, old: 28.9 ± 3.4 mV; *P* value for each data group >0.05). However, a slight decrease in mean AP half-width (APHW: young: 0.3 ± 0.01 ms; old: 0.26 ± 0.01 ms; *P* = 0.04) was, however, seen, possibly reflecting final maturation of the neuronal population. *N* = 5 GADnL neurons in young animals, 23 in old. Abbreviations: *R*_m_, input resistance; AP, action potential; APHW, action potential half-width; *I*_CMD_, step command current; *C*_m_, estimated neuronal capacitance (see methods). For averaged AP shapes, the thickness of the trace signifies ± SEM. Note that for correct comparison of current-to-firing relationships between neurons of possibly differing membrane resistances, the command current (ICMD) in bottom left panel is scaled by multiplying by with the measured *R*_m_ for each cell. Also note that in the bar graphs (bottom right), values for APHW and *I*_h_-sag have been scaled (100× and 10×, respectively) for visual clarity.

More interestingly, we found large numbers of patchable neurons in slices prepared in warm conditions from mice older than 6 weeks. Giga-seal formation in these cells was easier and recordings were more stable in both DCN and Purkinje neurons compared to recordings from cold-cut preparations. Therefore, it was not difficult to find and patch DCN neurons in slices cut at near-physiological temperature (**Figure 4**) from mice between 1 and 7 months of age, even though patching DCN neurons in slices from animals older than 4 weeks has been considered difficult enough to discourage research (Linnemann et al., 2006; Bagnall et al., 2009).

As an example of this newly discovered ability to conduct *in vitro* DCN patch-clamp experiments in adult animals, we performed a quick set of proof-of-principle experiments with DCN slices (cut at warm temperature) from juvenile/young adult (<P30) and mature (1 – 7 months) animals (**Figure 4**). Comparing the intrinsic properties of DCN neurons (*R*_m_, AP amplitude, half-width, threshold, AHP amplitude, or the amplitude of *I*_h_-like voltage “sag,” for details and values see legend for **Figure 4**) we found that these properties do not seem to change at-large as animals mature beyond the first month. Importantly, this finding supports the use of the same electrophysiological cell-type identification guidelines (Uusisaari and Knöpfel, 2010) for adult DCN neurons that are used for juvenile animals.

## DISCUSSION

Since invention of the acute brain slicing method, several studies have been conducted to enhance slice recovery after cutting (Lipton et al., 1995; Richerson and Messer, 1995; Brahma et al., 2000; Moyer and Brown, 2002; Ye et al., 2006), often by complex modifications of the composition of cutting solutions. Recently, Zhao et al. (2011) have reported that by combining brain embedding in agar and compression during slicing with post-dissection incubation in Na-free ACSF supplemented with ascorbate and thiourea, viable acute slices can be produced from mice aged 2–8 months. In the present work, no changes in the cutting solution have been employed as we have focused on comparing the effects of temperature during the cutting procedure. It is, however, possible or perhaps even likely that ACSF modifications specifically designed for the brain region of interest further improve slice quality by prolonging neuronal survival after cutting. Slice preparation at physiological temperature has been occasionally reported in the literature, (e.g., Zhang and Trussell, 1994; Person and Raman, 2011), but the present study is, to out knowledge, the first time that warm and cold dissection are compared.

Importantly, even though it would be premature to claim that acute slice quality from every brain region would benefit similarly from warm preparation temperature, there is no reason to assume that improvements should be limited to cerebellar slices. Although this work only includes results from two cerebellar neuronal classes, the advantages of warm cutting temperature have subsequently been confirmed in inferior olive, vestibular nuclei, accessory olfactory bulb, hippocampus, and cerebral cortex in mature mice as well as rats (Y. Yarom, personal communication). Due to the higher yield of healthy slices and simplification of the procedure by omission of the cooling steps, our laboratories have shifted from cold-cutting to warm-cutting for all slice preparations. We did not observe improvement in the already good quality of slices from juvenile (<P20) animals, but neither was there any evidence of the warm method being detrimental to juvenile slice health.

We can only speculate on the mechanism underlying the improvement in slice quality with normal temperatures. First, cooling the brains prior and during slicing procedure was originally seen as a means of slowing cellular metabolism and thereby supporting cell survival (Ivanov and Zilberter, 2011), as well as enhancing structural rigidity to support slicing (Edwards et al., 1989). However, with the development of high-quality vibrating slicers with minimal vertical vibration (such as the Campden Ci 700 smz), unnatural hardening of the tissue block is no longer necessary. Notably, no agar or other means are used to support the brain block during slicing. Importantly, we observed a strong negative relationship between the magnitude of vertical vibration in the blade and the quality of slices produced; we were unable to obtain good slices with a vibration magnitude of ≥ 0.5 μm when using warm solutions.

It should be noted that even though in the described method, brain removal and gluing were performed quickly, the entire cutting procedure takes longer than the conventional ice-cold cutting method due to the slower cutting speed. Occasionally, 10 min or longer is needed for the warm-cut procedure whereas completion of conventional cold-cut preparation with typically much faster cutting speeds would take only several minutes. However, we found that the long slicing duration is not damaging per se to the slices, as there is no significant difference in the quality of first versus last slices obtained from the same brain.

Although cooling slows cellular metabolism, activities of cell membrane transporters do not respond uniformly to temperature change, which can disrupt metabolic processes as well as ion-gradient homeostasis, impairing survival during and after slicing. This might pose fewer problems in juvenile animals, where the neurons are, in general, more resistant to various insults than in fully mature animals. Finally, it seems reasonable that warm, and therefore more fluid, lipid membranes are able to seal damage caused by cutting more efficiently than cold, rigid membranes (Drobnis et al., 1993).

In summary, neuronal properties are not degraded by lack of cooling during slicing. Furthermore, mature brain structures are less damaged by the cutting procedure when performed in warm rather than in cold temperatures. Taking into account the possibility that rapid cooling may result in aberrant and only partially reversible changes in intrinsic and network properties (Kirov et al., 1999,^2004^; Bourne et al., 2007), we suggest that acute brain preparation should, when possible, be performed in warm solutions, in order to prevent undesirable cold-shock-induced changes in the slices.

## Conflict of Interest Statement

The authors declare that the research was conducted in the absence of any commercial or financial relationships that could be construed as a potential conflict of interest.
